# Relation between number of teeth, malnutrition, and 3‐year mortality in elderly individuals ≥85 years

**DOI:** 10.1111/odi.14023

**Published:** 2021-09-27

**Authors:** Kensuke Nishio, Yasumichi Arai, Yukiko Abe, Michiyo Takayama, Mai Fukasawa, Daichi Oikawa, Tomoka Ito, Masaki Takatsu, Toshimitsu Iinuma

**Affiliations:** ^1^ Department of Complete Denture Prosthodontics Nihon University School of Dentistry Tokyo Japan; ^2^ Center for Supercentenarian Medical Research Keio University School of Medicine Tokyo Japan; ^3^ Center for Preventive Medicine Keio University School of Medicine Tokyo Japan

**Keywords:** GLIM criteria, life expectancy, malnutrition, oral health, very elderly

## Abstract

**Objective:**

The number of teeth has been shown to affect mortality. However, it is unclear why the number of teeth is associated with mortality. We focused on the number of teeth and malnutrition and examined whether these differences affect 3‐year all‐cause mortality among very elderly individuals.

**Methods:**

This analysis was conducted using data from the Tokyo Oldest Old Survey on Total Health study. Altogether 513 participants ≥85 years were categorized based on remaining teeth (0, 1–7, 8–18, ≥19). All‐cause mortality was determined by calculating the cumulative 3‐year survival rate according to the remaining number of teeth and the presence/absence of malnutrition. Further, hazard ratios (HRs) were analyzed using Cox regression analyses.

**Results:**

No difference was observed according to the number of teeth (*p* = 0.638), but the presence/absence of malnutrition was significantly associated with mortality (*p* < 0.001). Malnutrition was independently associated with higher HRs, even after adjusting for confounding factors associated with mortality. (HR: 2.315, 95% CI: 1.431–3.746). Additionally, adjusting for the number of teeth, HR remained significant (HR: 2.365, 95% CI: 1.449–3.853).

**Conclusion:**

In the very elderly, malnutrition—but not the number of teeth—was independently associated with 3‐year all‐cause mortality after adjusting for various health issues.

## 1 BACKGROUND

Current studies have shown that the global life expectancy continues to increase (Gulland, 2016; Kontis et al., 2017) considering that breakthroughs in medical technology have enabled the treatment of various diseases. Furthermore, given the improving nutritional condition and rising public health awareness (Costa, 2005; Cutler et al., 2006), most countries worldwide have an average life expectancy of 65 years or older, with some developed countries having an average life expectancy of 80 years or older (WHO, 2020).

Recently, geriatric epidemiology studies in the field of dentistry have identified a relationship between oral health, especially the number of teeth, and lifespan (Koka & Gupta, 2018). Accordingly, Hirotomi et al. reported significantly lower 5‐year mortality in 70‐year‐old subjects with a large number of remaining teeth when compared with those with a small number of remaining teeth (Hirotomi et al., 2015). Moreover, other studies have reported similar results (Goto et al., 2020; Holm‐Pedersen et al., 2008; Osterberg et al., 2008). As such, maintaining oral health, especially the number of teeth of elderly individuals carries numerous benefits. However, the reason why the number of teeth is a predictor of lifespan has not been described clearly in literature. It has been reported that the intake of various nutrients is lower when there are fewer remaining teeth (Nakamura et al., 2019), and it is presumed that malnutrition is involved.

Given that most previous reports had included individuals aged 80 or younger, extrapolating their findings to include the very elderly population would be difficult. In the very elderly, there is insufficient evidence of a relation between the number of teeth and mortality. As the average global life expectancy continues to increase, the next step would be to establish evidence for more elderly individuals. To date, our research team has conducted a longitudinal cohort study on participants aged 85 years and over wherein oral health and various factors were examined to accumulate evidence. We hypothesized that the number of teeth is associated with mortality, even in the very elderly, and that this is attributable to nutritional status. Therefore, the current study sought to explore the association between the number of teeth remaining in the oral cavity, malnutrition, and mortality among very elderly individuals to establish further evidence for this cohort.

## 2 METHODS

### 2.1 Participants

This study used data from the Tokyo Oldest Old Survey on Total Health (TOOTH) study. TOOTH was a longitudinal epidemiological survey of randomly selected very elderly people aged 85 years older residing in the Tokyo metropolitan area conducted between March 2008 and November 2009. All baseline data for this study were collected during that period. 542 participants who participated in an in‐home interview and clinical examination were enrolled in this study (Arai et al., 2010). After excluding 13 participants with missing data for the number of teeth, 4 participants with missing data for 3‐year mortality, and 12 participants with implant treatment, 513 participants were ultimately analyzed (women, *n* = 288; men, *n* = 225; age, 87.3 ± 2.3 years). The study was conducted after ethical approval from the Nihon University School of Dentistry (No. 2003–20, 2008) and Keio University School of Medicine (No. 20070047, 2007). The TOOTH survey was registered in the UMIN‐Clinical Trial Registry (ID UMIN000001842). The research aims and procedures were explained, and written informed consent was obtained either from participants or by proxy (normally a family member or caregiver) when individuals lacked the capacity to give a consent (Arai et al., 2010). This study was conducted in accordance with the STROBE Statement (Appendix, STROBE Checklist).

### 2.2 Oral health assessment

The oral health assessment comprised an oral examination, face‐to‐face interview, and questionnaire regarding oral health behaviors by trained dentists (Iinuma et al., 2012). An oral examination of the remaining teeth (range 0–32) and calculation of the maximum occlusal force (MOF) were performed by qualified dentists during face‐to‐face interviews. MOF was defined as the maximum bite force during voluntary clenching, considered as an index of oral function, and measured with an Occlusal Force‐Meter (GM10; Nagano Keiki, Inc.). Measurement of MOF was performed at the first molars bilaterally. In patients wearing dentures, the prostheses were inserted into the mouth during MOF measurement (Iinuma et al., 2016). Oral health‐related quality of life was evaluated using the Geriatric Oral Health Assessment Index (GOHAI) (Naito et al., 2006), based on a total score of 60 points for 12 questions. There were also questionnaires about denture; we asked about the frequency of denture use and the complaints about the dentures for denture wearers. Both questions were answered on a scale of 5 (1, never; 2, seldom; 3, sometimes; 4, often; 5, always). For the question on frequency, participants who responded with a 4 or 5 were defined as “frequency of use dentures (almost every day).” For the question on presence or absence of complaints about dentures, participants who answered 3–5 were defined “having complaints of dentures.”

### 2.3 Demographics and general health assessment

Relevant demographic and general health information was collected during face‐to‐face interviews that included age, gender, household details, education, tobacco smoking and drinking habits, cognitive function, and systemic illness. Smoking and drinking were dichotomized in terms of whether participants had never or had ever smoked and whether they had ever or had never consumed alcohol, respectively. The household composition was evaluated based on whether they lived alone. Education level was recorded as a binary variable, that is, whether they had completed high school. Activities of Daily Living (ADL) was evaluated using the Barthel Index (Mahoney & Barthel, 1965), and a disability in ADL was defined as having a disability on one or more indices. The calf circumference was measured twice, and the average value was used. Participants’ cognitive state was graded using the Mini‐Mental State Examination (MMSE) (Folstein et al., 1975), and the percentage of participants with a score <24, defined as the suspected cognitive impairment (Shibasaki et al., 2018; Trzepacz et al., 2015), was calculated. Body mass index (BMI) was calculated from the height and weight measurements. The World Health Organization five well‐being index (WHO‐5) was used to assess psychological status. Using non‐fasting blood samples, C‐reactive protein was measured by standard assay procedures. Existing medical conditions were coded with the International Classification of Diseases, 10th revision.

During medical history evaluation, information on cardiovascular diseases was gathered, including stroke, transient ischemic attack, angina, and myocardial infarction. Hypertension was considered when the systolic blood pressure was recorded >140 mmHg during baseline examination or if the patient was currently using antihypertensive medications. Considering the high prevalence of undiagnosed diabetes mellitus in old age, it was confirmed when at least one of the following criteria was fulfilled: (1) self‐reported diagnosis, (2) use of insulin or other oral antidiabetic drugs, (3) random blood sugar ≥200 mg/dl, or (4) glycated hemoglobin (HbA1c) ≥6.5%.

### 2.4 Physical performance survey

Handgrip strength was measured with a handheld dynamometer (Tanita 6103; Tanita Corp.) on the dominant hand. The average of two measurements was calculated. In contrast, the timed up and go test measured the motor function of the lower extremity (Podsiadlo & Richardson, 1991).

### 2.5 Definition of malnutrition

There are several criteria for malnutrition. Nutritional status reflects the characteristics of various regions. Therefore, universal diagnostic criteria have been needed. In this study, we defined malnutrition using the Global Leadership Initiative on Malnutrition (GLIM) criteria (Table S1) (Cederholm et al., 2019; Jensen et al., 2019). This is a new and international criterion. We applied our data to the GLIM criteria to calculate which participants had malnutrition. In non‐volitional weight loss, we substituted our questionnaire results concerning “weight loss of over 3 kg in a year.” In reduced muscle mass, we substituted our measurements of calf circumference. The cutoff followed the Asian consensus (Chen et al., 2020). For reduction of food intake, we substituted GOHAI question 1 (How often do you limit the kinds or amounts of food you eat because of problems with your oral condition?) (Naito et al., 2006). If the answer to this question was 1 (always) and 2 (often), it indicated a reduction in food intake.

### 2.6 Outcomes

This study focused on differences in mortality according to the number of teeth and presence or absence the malnutrition in very elderly individuals. Mortality was determined by a regular telephone call or email survey every 12 months. We performed it three times during the period.

### 2.7 Statistical analysis

The collected data were imported to SPSS ver. 26.0 (SPSS, Chicago, IL, USA) for statistical analysis. For examining the effect of the number of teeth in detail, the participants in this study were divided into four categories. These categories were determined based on the number of participants with edentulous and on the tertiles of the number of participants with dentulous (0; 1–7; 8–18; ≥19). Descriptive data were summarized as mean with standard deviation (SD) or median with interquartile range (IQR) for continuous variables and frequency (percentages) for categorical variables. Differences among the four categories were assessed using analysis of variance, the Kruskal–Wallis test for evaluating continuous variables, and the chi‐square test for categorical variables. Kaplan–Meier plots were used to display survival curves according to the number of teeth (four categories) and presence or absence of malnutrition. Survival rates were compared using the log‐rank test. Hazard ratios (HRs) and 95% confidence intervals (CIs) were estimated using Cox regression analyses.

The following covariates, which are commonly associated with mortality, were selected as confounders: sex (categorical: male/female), age (continuous), living alone, ADL disability, MMSE (<24), handgrip strength, and medical history (cancer and diabetes). Thereafter, three models were created for analysis. Model 1 was adjusted for sex and age. Model 2 was adjusted for all selected confounding factors. Model 3 added the effect of the number of teeth (four categories) or presence or absence of malnutrition. Because the number of missing data varies depending on the confounders, we performed the analysis on participants whose data were available in each model. All tests were two‐sided, with a statistical confidence of 5%.

## 3 RESULTS

The number of participants according to the four categories was as follows: (1) edentulous (*n* = 157), (2) 1–7 (*n* = 124), (3) 8–18 (*n* = 120), and (4) ≥19 (*n* = 112). Baseline characteristics of participants according to the number of teeth are summarized in Table 1. Accordingly, the number of teeth was significantly associated with age, ADL disability, MMSE, handgrip strength, diabetes mellitus, cancer, MOF, GOHAI, and denture wearing. In the item of denture wearing, about 90% of participants wore dentures. Unexpectedly, the number of teeth was not associated with malnutrition and questions about dentures.

**TABLE 1:** Baseline characteristics of participants according to number of teeth (four categories)

	Number of teeth (4 categories)					
	all	0	1–7	8–18	≥19	p*
Characteristics	513	157	124	120	112	
Demographics						
Sex (% female)	56	63	58	54	46	0.052
Age (y), mean (*SD*)	87.3 (2.3)	87.8 (2.6)	87.4 (2.4)	86.8 (1.9)	87.1 (1.8)	0.001
Higher education (%)^b^	25	20	24	26	31	0.270
Smoking (%)^c^	39	36	40	40	43	0.715
Drinking (%)^d^	47	41	45	50	54	0.178
Living alone (%)^c^	34	32	38	28	37	0.314
General health assessment						
Body mass index (kg/m^2^), mean (SD)^e^	21.4 (3.2)	21.2 (3.3)	21.7 (3.2)	21.3 (2.7)	21.6 (3.3)	0.448
WHO−5, median (IQR)^f^	19 (15–22)	19 (15–23)	20 (15–23)	18 (14–22)	20 (16–23)	0.156^a^
ADL disability (%)^g^	27	32	35	24	14	0.001
Calf Circumference (cm), mean (SD)^h^	32.0 (3.2)	31.5 (3.3)	32.1 (3.1)	32.3 (3.0)	32.5 (3.3)	0.129
Weight loss (%)^i^	17	20	19	16	9	0.129
MMSE <24 (%)^h^	21	20	23	26	12	0.047
Physical status						
Handgrip strength (kg), mean (SD)^j^	19.6 (5.8)	18.6 (5.5)	19.3 (5.9)	19.9 (5.1)	20.8 (6.3)	0.017
Timed up and go test, median (IQR)^k^	13.1 (10.9–16.2)	13.5 (10.9–16.9)	13.3 (11.7–16.7)	12.2 (10.3–15.3)	13.4 (11.4–15.5)	0.062^a^
Medical history (%)						
Hypertension	82	79	82	85	84	0.705
Diabetes mellitus	16	16	17	23	7	0.015
Cardiovascular disease	22	25	22	19	22	0.732
Cancer	19	18	20	13	28	0.031
Biochemical						
CRP, mg/dl, median (IQR)^l^	0.09 (0.04–0.18)	0.09 (0.04–0.20)	0.10 (0.05–0.19)	0.09 (0.04–0.18)	0.08 (0.04–0.17)	0.492^a^
Dental and oral health status						
Maximum Occlusal Force, median (IQR)^m^	11.7 (6.6–19.2)	7.8 (4.1–12.5)	9.7 (6.3–15.1)	11.4 (7.4–19.2)	21.9 (15.4–32.8)	<0.001^a^
GOHAI, median (IQR)^j^	56 (51–58)	54 (50–58)	55 (50–58)	54 (50–58)	58 (55–60)	<0.001^a^
Denture wearer (%)	88	99	100	97	51	<0.001
Denture questions (denture wearers only)						
Frequency of use dentures						
Almost every day (%)^n^	94	93	95	95	91	0.704
Having complaints of denture (%)^o^	31	35	31	31	18	0.158
Nutritional status						
Reduction of food intake (%)^p^	12	12	15	10	12	0.730
Malnutrition (%)	17	17	24	16	13	0.159

Note

*p‐values were calculated for categorical covariates using chi‐square test, whereas *p*‐values were calculated using one‐way analysis of variance for continuous variables unless otherwise indicated. ^a^Calculated using nonparametric Kruskal–Wallis test. ^b–q^Data available for ^b^493, ^c^500, ^d^498, ^e^500, ^f^510, ^g^497, ^h^505, ^i^506, ^j^511, ^k^435, ^l^509, ^m^476, ^n^453, 445, ^p^501 people, respectively.

Abbreviations: ADL, Activities of Daily Living; CRP, C‐reactive protein; GOHAI, Geriatric Oral Health Assessment indexIQR, interquartile range; MMSE, Mini‐Mental State Examination; SD, standard deviation; WHO‐5, The five‐item World Health Organization Well‐Being Index.

### 3.1 Number of teeth and 3‐year all‐cause mortality

**FIGURE 1:**
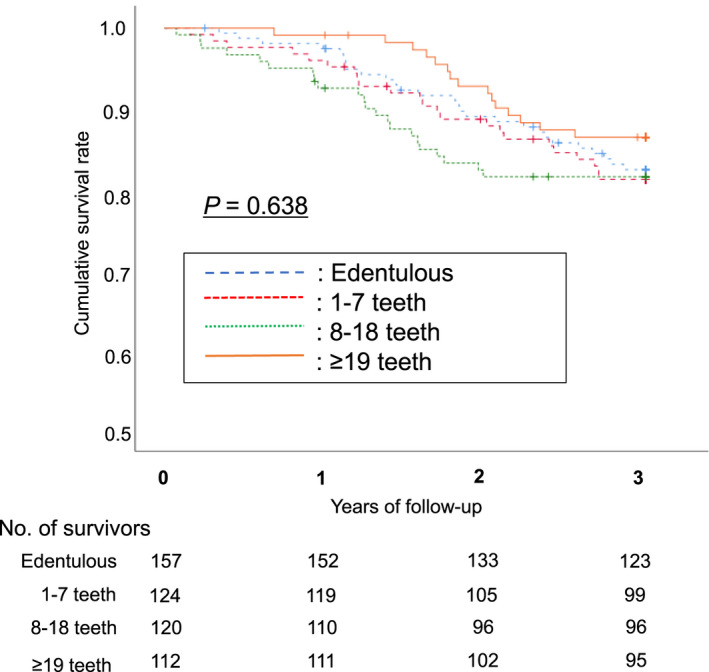
Number of teeth and 3‐year all‐cause mortality. Kaplan–Meier survival curves for the four categories of number of teeth (log‐rank *p* = 0.638). Cumulative survival rates did not differ due to differences in the number of teeth

Survival analysis during the 3‐year follow‐up period showed that 89 participants died. 18 participants dropped out during the period, and their data were used to calculate mortality until they dropped out. Kaplan–Meier survival curves for the cumulative incidence of mortality (Figure 1) showed no significant differences between all categories of tooth count (log‐rank *p* = 0.638).

### 3.2 Malnutrition and 3‐year all‐cause mortality

**FIGURE 2:**
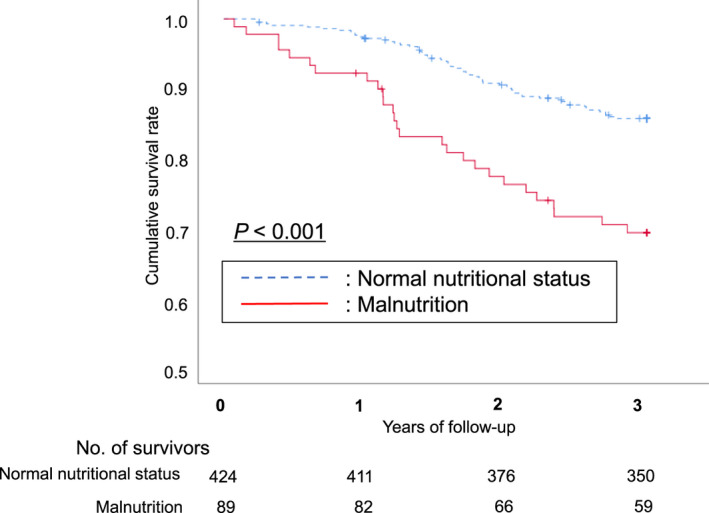
Malnutrition and 3‐year all‐cause mortality. Kaplan–Meier survival curves for the presence or absence of malnutrition. Cumulative survival rates differed (log‐rank *p* < 0.001)

Likewise, we plotted Kaplan–Meier survival curves for the cumulative incidence of mortality (Figure 2). The curve showed that there was a significant difference in the presence or absence of malnutrition (*p *< 0.001). The results of Cox regression analysis are shown in Table 2. After adjusting the several confounding factors, malnutrition was associated with more than twice higher risk of 3‐year mortality (Model 1, HR: 2.288, 95% CI: 1.449–3.614. Model 2, HR: 2.315, 95% CI: 1.431–3.746, respectively). After adjusting for the effects of the number of teeth (four categories), significant differences were still observed (HR: 2.365, 95% CI: 1.449–3.853) was observed.

**TABLE 2:** Hazard ratios for presence or absence of the malnutrition

	Model 1 (*n* = 513)			Model 2 (*n* = 490)			Model 3 (*n* = 490)		
	HR	95% Cl	*p*‐value	HR	95% Cl	*p*‐value	HR	95% Cl	*p*‐value
Malnutrition	2.288	[1.449–3.614]	<0.001	2.315	[1.431–3.746]	0.001	2.365	[1.449–3.853]	0.001

Note

Model 1: Adjusted for age, and gender. Model 2: Adjusted for age, gender, living alone, ADL disability, MMSE, handgrip strength, and medical history (cancer and diabetes). Model 3: Adjusted for Model 2 and the number of teeth (four categories).

Abbreviations: ADL, Activities of Daily Living; Cl, confidence interval; HR, hazard ratio; MMSE, Mini‐Mental State Examination.

**FIGURE 3:**
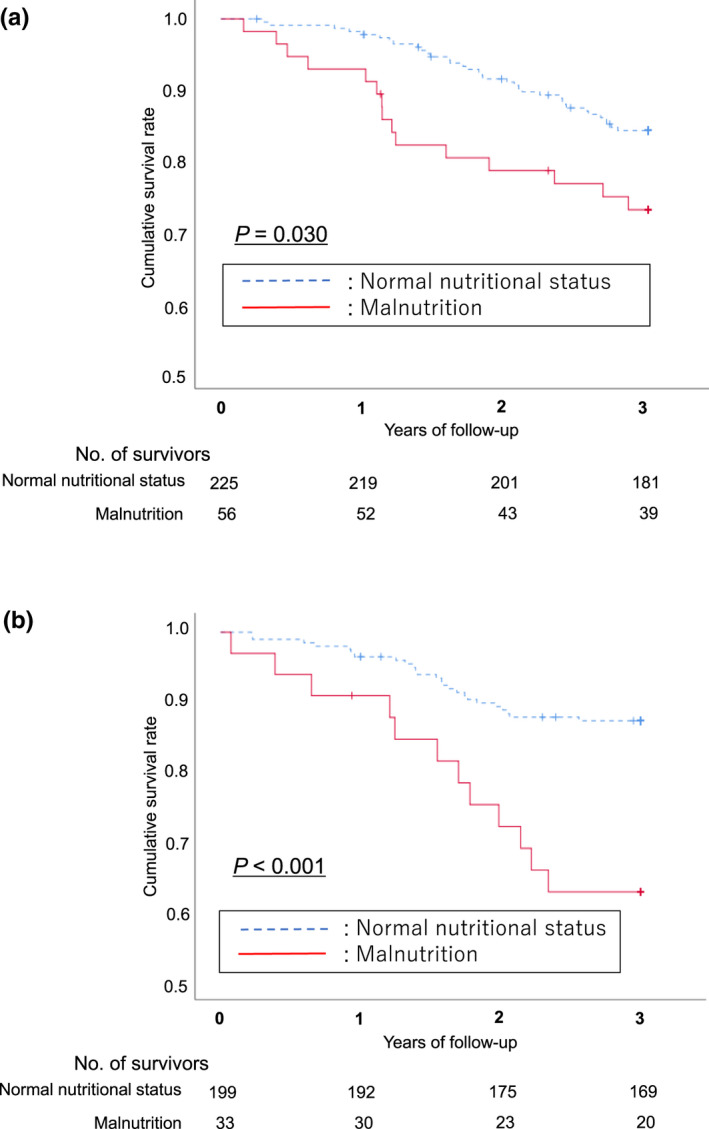
Kaplan–Meier survival curves for the presence or absence of malnutrition and 3‐year all‐cause mortality in two categories of teeth. A, 0–7 teeth. Cumulative survival rates differed (log‐rank *p* = 0.030). B, ≥8 teeth. Cumulative survival rate differed (log‐rank *p* < 0.001)

Additionally, the participants were divided into two groups according to the first and second half of the four categories (0–7 teeth/≥8 teeth), and the relationship between the presence or absence of malnutrition and the 3‐year mortality was examined (Figure 3). Differences were also observed in both groups with and without malnutrition (log‐rank *p* = 0.030, <0.001).

## 4 DISCUSSION

Contrary to our hypothesis, the current study on very elderly individuals aged ≥85 years found no significant difference in 3‐year mortality according to the number of teeth. However, we found a significant difference according to the presence or absence of malnutrition. Further, malnutrition independently affected HR after adjusting for several confounding factors, including the number of teeth.

Several studies reported that an increased number of remaining teeth was associated with mortality (Goto et al., 2020; Hirotomi et al., 2015; Holm‐Pedersen et al., 2008; Osterberg et al., 2008). However, Ando et al. reported that there was no association of number of teeth with all‐cause mortality among men aged 65–79 years (Ando et al., 2014), and our result was similar to that study. We also analyzed between the two groups using a 20‐tooth cutoff, which was allowed in some literature and found no significant difference in mortality (log‐rank *p* = 0.497, Figure S1). The participants in the current study were over 85 years, with the oldest being 100 years old. The number of teeth decreases with age. The median number of teeth in current study was 8. Compared to previous literature that examined the number of teeth and mortality, the number of at baseline was low in this population, and it was possible that the number of teeth had less of an effect in people older than 85 years, and that there may be other strong risk factors for mortality.

In contrast, the presence or absence of malnutrition showed a difference in the 3‐year mortality of the participants, and HR was more than doubled in the presence of malnutrition, even after adjusting for various confounding factors. Previous reports had also reported a link between nutritional status and mortality (Jayanama et al., 2018; Malafarina et al., 2018). Although many reports were not limited to participants ≥85 years old, the results of this study were compatible with those reports. Therefore, the current study confirmed that malnutrition is a risk factor for mortality even in the very elderly. In this study, we focused on comprehensive nutritional status and not on individual nutrient intake, such as proteins and vitamins. As Osawa et al. reported (Osawa et al., 2017), there were a wide variety of dietary patterns. It is unlikely that only one nutrient will deviate significantly from the reference value. Because various nutrients are ingested from one food, it is more clinically realistic to focus on malnutrition overall than to focus on malnutrition of individual nutrients.

It is not clear why the number of teeth did not affect the 3‐year mortality, but it may be because there was no association between the number of teeth and the percentage of participants with low nutrition in this study. From the results in Table 1, the number of malnourished participants was the smallest in the category with ≥19 teeth, but no significant difference was observed among all categories. The results of malnutrition and HRs still showed significant differences, even when considering the effects of the number of teeth. Based on this result, it was found that the number of teeth was not related to malnutrition in this group of very elderly participants. We also analyzed MOF and GOHAI as confounders instead of the number of teeth, but there was no change (Table S2). We also found a significant difference in 3‐year mortality depending on the presence or absence of malnutrition in the categories of a large and small number of teeth, those with a small number of teeth can live a long life if malnutrition can be prevented.

Study of the relationship between oral health, including the number of teeth, and malnutrition in this participant in detail is warranted in the future. There have been few large cohorts to date that have examined oral health and nutritional status in detail. Although the results of our study cannot be directly compared with those of other cohorts, there are several possible reasons why we did not find a difference in the rate of malnutrition according to the number of teeth. An association between occlusal support and nutrient intake has been reported, but our data did not show any difference in the rate of malnutrition between different Eichner Index (*p* = 0.589, table S3). It can be inferred that these factors other than the number of teeth and occlusal support are important in the development of malnutrition. Of the participants in the current study, approximately 90% wore dentures. It has been reported that patients who are edentulous and without dentures have a shorter survival (Sabbah et al., 2020). This may also apply to patients who wear dentures that are similar in form to complete dentures. Although the form of the denture is different for each participant, we conducted a questionnaire survey on the complaints of the denture and the wearing time of the denture. If the number of remaining teeth is reduced, the denture form will become larger and the treatment will be difficult (Gilbert et al., 2004). However, our results showed no difference between the two denture questionnaires for frequency and complaints, regardless of the number of teeth. This result suggested that the dentures of the participants were functioning to some extent regardless of the number of teeth. Furthermore, in GOHAI, there was a difference in the total score due to the difference in the number of teeth, but in the report of 2005 census of Japan, the median of GOHAI in the 70s was reported as 52.8. In our results, even the group of edentulous participants exceeded that value (edentulous median, 55) (MIC, 2005). For these reasons, it was possible that the participants in the current study were a group in which oral function was maintained to some extent, and we believe that the quality of the prosthetic appliances is associated with malnutrition. Moreover, the number of teeth may not be an important factor in mortality, even if the number of teeth is low, as long as the prosthesis, such as dentures, is functioning properly. To elucidate this hypothesis, we need to investigate further the oral health factors associated with malnutrition in the very old.

There are some limitations to this study. First, in this study, 12 participants who received dental implants were excluded; therefore, patients who received dental implants were not taken into account. The breakdown of treatment for these 12 participants was as follows: 8 had fixed implants (one had bone‐anchored bridge) and 4 had implant overdentures. As there is no uniformity in the prosthetics of a participant who has undergone implant treatment and the effect of the number of teeth on this study outcome may be not accurately reflected through this, participants who received implant treatment were excluded. Because the number of elderly people treated with implants is expected to increase in the future, further epidemiological studies that take into account the impact of implants are required. Second, the number of participants and the areas covered under research were limited, because our study targeted very elderly people who lived in the heart of Tokyo. Therefore, a larger‐scale, separate cohort study that considers race, eating habits, and economic conditions should be conducted. Furthermore, to elucidate the biological mechanisms underlying the relationship between the number of teeth, malnutrition, and mortality, investigating the cause‐specific mortality of each is essential. However, in this study, we did not have sufficient participants to analyze cause‐specific mortality. Third, in the analysis of the presence of malnutrition and the survival rate after 3 years, the power [0.05 alpha (two‐sided)] was sufficient score of 0.946 for all participants (survival rate: 85%normal nutritional status, 69% malnutrition). However, in the subgroup analysis, the power for the group with ≥8 teeth was 0.895 (survival rate: 87% normal nutritional status, 63% malnutrition), whereas the power for the group with 0–7 teeth was 0.356 (survival rate: 84% normal nutritional status, 74% malnutrition). Therefore, the number of participants may have been small in the subgroup analysis, and further large‐scale studies are needed in the future. Finally, we reported the results of the 3‐year follow‐up of the study. Our results showed that the number of teeth was associated with ADL disability. Since ADL is related to mortality (Nakazawa et al., 2012), a longer observation period might have made a difference in the number of teeth and mortality; however, we conducted this study to accumulate further evidence concerning the very elderly. Information obtained via analysis using data from very elderly healthy participants is extremely rare and is essential for establishing new aging evidence. We aimed to further enhance the lives of the elderly by clarifying the relationship between factors, such as oral health, malnutrition, and mortality, which were not evaluated in previous reports in this age group. Finally, the importance of maintaining the number of teeth remains the same for the very elderly. It is a general concept in gerodontology that the number of teeth is related to frailty (Hakeem et al., 2019; Tôrres et al., 2015). Our report does not conclude that keeping the number of teeth is ineffective. It showed that mortality is not impaired if malnutrition can be avoided even with a small number of teeth, suggesting that the number of teeth may not be an absolute predictor of mortality in this group of elderly.

## 5 CONCLUSION

In a cohort of very elderly people, there was no difference in 3‐year mortality due to the difference in the number of remaining teeth. However, malnutrition affected 3‐year mortality. Furthermore, malnutrition independently affected HR, even after adjusting for several confounding factors, including the number of teeth. Among the participants in this study, there was no significant difference in the incidence of malnutrition based on the number of teeth. These results suggest that even for those with few teeth, longevity will not be affected if they can get moderate nutrition. Our results suggest that the number of teeth may not be an absolute predictor of mortality in this group of the very elderly.

## CONFLICT OF INTEREST

The authors report no conflicts of interest related to this work.

## AUTHOR CONTRIBUTIONS

**Kensuke Nishio:** Conceptualization; Data curation; Formal analysis; Investigation; Methodology; Visualization; Writing‐original draft; Writing‐review & editing. **Yasumichi Arai:** Conceptualization; Data curation; Formal analysis; Funding acquisition; Investigation; Methodology; Project administration; Supervision; Writing‐original draft; Writing‐review & editing. **Yukiko Abe:** Data curation; Formal analysis; Investigation; Methodology; Writing‐review & editing. **Michiyo Takayama:** Data curation; Formal analysis; Investigation; Methodology; Writing‐review & editing. **Mai Fukasawa:** Writing‐review & editing. **Daichi Oikawa:** Writing‐review & editing. **Tomoka Ito:** Writing‐review & editing. **Masaki Takatsu:** Writing‐review & editing. **Toshimitsu Iinuma:** Conceptualization; Data curation; Formal analysis; Funding acquisition; Investigation; Methodology; Project administration; Supervision; Writing‐original draft; Writing‐review & editing.

### PEER REVIEW

The peer review history for this article is available at https://publons.com/publon/10.1111/odi.14023.

## Supporting information

Fig S1

Table S1‐3

Supplementary Material

## Data Availability

Author elects to not share data.

## References

[odi14023-bib-0001] Ando, A. , Tanno, K. , Ohsawa, M. , Onoda, T. , Sakata, K. , Tanaka, F. , Makita, S. , Nakamura, M. , Omama, S. , Ogasawara, K. , Ishibashi, Y. , Kuribayashi, T. , Koyama, T. , Itai, K. , Ogawa, A. , & Okayama, A. (2014). Associations of number of teeth with risks for all‐cause mortality and cause‐specific mortality in middle‐aged and elderly men in the northern part of Japan: The Iwate‐KENCO study. Community Dentistry and Oral Epidemiology, 42(4), 358–365. 10.1111/cdoe.12095 24476489

[odi14023-bib-0002] Arai, Y. , Iinuma, T. , Takayama, M. , Takayama, M. , Abe, Y. , Fukuda, R. , Ando, J. , Ohta, K. , Hanabusa, H. , Asakura, K. , Nishiwaki, Y. , Gondo, Y. , Akiyama, H. , Komiyama, K. , Gionhaku, N. , & Hirose, N. (2010). The Tokyo Oldest Old survey on Total Health (TOOTH): A longitudinal cohort study of multidimensional components of health and well‐being. BMC Geriatrics, 10, 35. 10.1186/1471-2318-10-35 20529368 PMC2893189

[odi14023-bib-0003] Cederholm, T. , Jensen, G. L. , Correia, M. , Gonzalez, M. C. , Fukushima, R. , Higashiguchi, T. , Baptista, G. , Barazzoni, R. , Blaauw, R. , Coats, A. J. S. , Crivelli, A. N. , Evans, D. C. , Gramlich, L. , Fuchs‐Tarlovsky, V. , Keller, H. , Llido, L. , Malone, A. , Mogensen, K. M. , Morley, J. , … Compher, C. (2019). GLIM criteria for the diagnosis of malnutrition ‐ A consensus report from the global clinical nutrition community. Journal of Cachexia, Sarcopenia, and Muscle, 10(1), 207–217. 10.1002/jcsm.12383 30920778 PMC6438340

[odi14023-bib-0004] Chen, L. K. , Woo, J. , Assantachai, P. , Auyeung, T. W. , Chou, M. Y. , Iijima, K. , Jang, H. C. , Kang, L. , Kim, M. , Kim, S. , Kojima, T. , Kuzuya, M. , Lee, J. S. W. , Lee, S. Y. , Lee, W.‐J. , Lee, Y. , Liang, C.‐K. , Lim, J.‐Y. , Lim, W. S. , … Arai, H. (2020). Asian working group for sarcopenia: 2019 consensus update on sarcopenia diagnosis and treatment. Journal of the American Medical Directors Association, 21(3), 300–307.e302. 10.1016/j.jamda.2019.12.012 32033882

[odi14023-bib-0005] Costa, D. L. (2005). Causes of improving health and longevity at older ages: a review of the explanations. GENUS, LXI (No. 1), 61(1), 21–28. https://www.jstor.org/stable/29788834

[odi14023-bib-0006] Cutler, D. , Deaton, A. , & Lleras‐Muney , A. (2006). The determinants of mortality. Journal of Economic Perspectives, 20, 97–120. 10.1257/jep.20.3.97

[odi14023-bib-0007] Folstein, M. F. , Folstein, S. E. , & McHugh, P. R. (1975). "Mini‐mental state". A practical method for grading the cognitive state of patients for the clinician. Journal of Psychiatric Research, 12(3), 189–198. 10.1016/0022-3956(75)90026-6 1202204

[odi14023-bib-0008] Gilbert, G. H. , Meng, X. , Duncan, R. P. , & Shelton, B. J. (2004). Incidence of tooth loss and prosthodontic dental care: Effect on chewing difficulty onset, a component of oral health‐related quality of life. Journal of the American Geriatrics Society, 52(6), 880–885. 10.1111/j.1532-5415.2004.52253.x 15161450

[odi14023-bib-0009] Goto, Y. , Wada, K. , Uji, T. , Koda, S. , Mizuta, F. , Yamakawa, M. , & Nagata, C. (2020). Number of teeth and all‐cause and cancer mortality in a Japanese community: The Takayama Study. Journal of Epidemiology, 30(5), 213–218. 10.2188/jea.JE20180243 31006716 PMC7153964

[odi14023-bib-0010] Gulland, A. (2016). Global life expectancy increases by five years. BMJ, 353, i2883. 10.1136/bmj.i2883 27197748

[odi14023-bib-0011] Hakeem, F. F. , Bernabé, E. , & Sabbah, W. (2019). Association between oral health and frailty: A systematic review of longitudinal studies. Gerodontology, 36(3), 205–215. 10.1111/ger.12406 31025772

[odi14023-bib-0012] Hirotomi, T. , Yoshihara, A. , Ogawa, H. , & Miyazaki, H. (2015). Number of teeth and 5‐year mortality in an elderly population. Community Dentistry and Oral Epidemiology, 43(3), 226–231. 10.1111/cdoe.12146 25600364

[odi14023-bib-0013] Holm‐Pedersen, P. , Schultz‐Larsen, K. , Christiansen, N. , & Avlund, K. (2008). Tooth loss and subsequent disability and mortality in old age. Journal of the American Geriatrics Society, 56(3), 429–435. 10.1111/j.1532-5415.2007.01602.x 18194226

[odi14023-bib-0014] Iinuma, T. , Arai, Y. , Fukumoto, M. , Takayama, M. , Abe, Y. , Asakura, K. , Nishiwaki, Y. , Takebayashi, T. , Iwase, T. , Komiyama, K. , Gionhaku, N. , & Hirose, N. (2012). Maximum occlusal force and physical performance in the oldest old: the Tokyo oldest old survey on total health. Journal of the American Geriatrics Society, 60(1), 68–76. 10.1111/j.1532-5415.2011.03780.x 22211666

[odi14023-bib-0015] Iinuma, T. , Arai, Y. , Takayama, M. , Abe, Y. , Ito, T. , Kondo, Y. , Hirose, N. , & Gionhaku, N. (2016). Association between maximum occlusal force and 3‐year all‐cause mortality in community‐dwelling elderly people. BMC Oral Health, 16(1), 82. 10.1186/s12903-016-0283-z 27586200 PMC5009498

[odi14023-bib-0016] Jayanama, K. , Theou, O. , Blodgett, J. M. , Cahill, L. , & Rockwood , K. (2018). Frailty, nutrition‐related parameters, and mortality across the adult age spectrum. BMC Medicine, 16(1), 188. 10.1186/s12916-018-1176-6 30360759 PMC6202862

[odi14023-bib-0017] Jensen, G. L. , Cederholm, T. , Correia, M. I. T. D. , Gonzalez, M. C. , Fukushima, R. , Higashiguchi, T. , Baptista, G. A. , Barazzoni, R. , Blaauw, R. , Coats, A. J. S. , Crivelli, A. , Evans, D. C. , Gramlich, L. , Fuchs‐Tarlovsky, V. , Keller, H. , Llido, L. , Malone, A. , Mogensen, K. M. , Morley, J. E. , … Gossum, A. (2019). GLIM criteria for the diagnosis of malnutrition: A Consensus Report from the global clinical nutrition community. JPEN: Journal of Parenteral and Enteral Nutrition, 43(1), 32–40. 10.1002/jpen.1440 30175461

[odi14023-bib-0018] Koka, S. , & Gupta, A. (2018). Association between missing tooth count and mortality: A systematic review. Journal of Prosthodontic Research, 62(2), 134–151. 10.1016/j.jpor.2017.08.003 28869174

[odi14023-bib-0019] Kontis, V. , Bennett, J. E. , Mathers, C. D. , Li, G. , Foreman, K. , & Ezzati, M. (2017). Future life expectancy in 35 industrialised countries: Projections with a Bayesian model ensemble. The Lancet, 389(10076), 1323–1335. 10.1016/s0140-6736(16)32381-9 PMC5387671 28236464

[odi14023-bib-0020] Mahoney, F. I. , & Barthel, D. W. (1965). Functional evaluation: The Barthel Index. Maryland State Medical Journal, 14, 61–65. 14258950

[odi14023-bib-0021] Malafarina, V. , Reginster, J. Y. , Cabrerizo, S. , Bruyère, O. , Kanis, J. A. , Martinez, J. A. , & Zulet, M. A. (2018). Nutritional status and nutritional treatment are related to outcomes and mortality in older adults with hip fracture. Nutrients, 10(5), 555. 10.3390/nu10050555 29710860 PMC5986435

[odi14023-bib-0022] Ministry of Internal Affairs and Communications . (2005). National consensus in 2005. Retrived December 1, 2020, from https://www.qualitest.jp/qol/files/gohai_norm.pdf

[odi14023-bib-0023] Naito, M. , Suzukamo, Y. , Nakayama, T. , Hamajima, N. , & Fukuhara, S. (2006). Linguistic adaptation and validation of the General Oral Health Assessment Index (GOHAI) in an elderly Japanese population. Journal of Public Health Dentistry, 66(4), 273–275. 10.1111/j.1752-7325.2006.tb04081.x 17225823

[odi14023-bib-0024] Nakamura, M. , Ojima, T. , Nagahata, T. , Kondo, I. , Ninomiya, T. , Yoshita, K. , Arai, Y. , Ohkubo, T. , Murakami, K. , Nishi, N. , Murakami, Y. , Takashima, N. , Okuda, N. , Kadota, A. , Miyagawa, N. , Kondo, K. , Okamura, T. , Ueshima, H. , Okayama, A. , … Miura, K. (2019). Having few remaining teeth is associated with a low nutrient intake and low serum albumin levels in middle‐aged and older Japanese individuals: Findings from the NIPPON DATA2010. Environmental Health and Preventive Medicine, 24(1), 1. 10.1186/s12199-018-0752-x 30611201 PMC6320628

[odi14023-bib-0025] Nakazawa, A. , Nakamura, K. , Kitamura, K. , & Yoshizawa, Y. (2012). Association between activities of daily living and mortality among institutionalized elderly adults in Japan. Journal of Epidemiology, 22(6), 501–507. 10.2188/jea.je20110153 22850544 PMC3798561

[odi14023-bib-0026] Osawa, Y. , Arai, Y. , Takayama, M. , Hirata, T. , Kawasaki, M. , Abe, Y. , Iinuma, T. , Sasaki, S. , & Hirose, N. (2017). Identification of dietary patterns and their relationships with general and oral health in the very old. Asia Pacific Journal of Clinical Nutrition, 26(2), 262–270. 10.6133/apjcn.022016.02 28244704

[odi14023-bib-0027] Osterberg, T. , Carlsson, G. E. , Sundh, V. , & Mellström, D. (2008). Number of teeth–a predictor of mortality in 70‐year‐old subjects. Community Dentistry and Oral Epidemiology, 36(3), 258–268. 10.1111/j.1600-0528.2007.00413.x 18474058

[odi14023-bib-0028] Podsiadlo, D. , & Richardson, S. (1991). The timed “Up & Go”: A test of basic functional mobility for frail elderly persons. Journal of the American Geriatrics Society, 39(2), 142–148. 10.1111/j.1532-5415.1991.tb01616.x 1991946

[odi14023-bib-0029] Sabbah, W. , Slade, G. D. , Sanders, A. E. , & Bernabé, E. (2020). Denture wearing and mortality risk in edentulous American adults: A propensity score analysis. Journal of Dentistry, 100, 103360. 10.1016/j.jdent.2020.103360 32404256

[odi14023-bib-0030] Shibasaki, K. , Asahi, T. , Mizobuchi, K. , Akishita, M. , & Ogawa, S. (2018). Rehabilitation strategy for hip fracture, focused on behavioral psychological symptoms of dementia for older people with cognitive impairment: A nationwide Japan rehabilitation database. PLoS One, 13(7), e0200143. 10.1371/journal.pone.0200143 29975757 PMC6033436

[odi14023-bib-0031] Tôrres, L. H. , Tellez, M. , Hilgert, J. B. , Hugo, F. N. , de Sousa, M. D. , & Ismail, A. I. (2015). Frailty, frailty components, and oral health: A systematic review. Journal of the American Geriatrics Society, 63(12), 2555–2562. 10.1111/jgs.13826 26563844

[odi14023-bib-0032] Trzepacz, P. T. , Hochstetler, H. , Wang, S. , Walker, B. , & Saykin, A. J. (2015). Relationship between the Montreal cognitive assessment and Mini‐mental State examination for assessment of mild cognitive impairment in older adults. BMC Geriatrics, 15, 107. 10.1186/s12877-015-0103-3 26346644 PMC4562190

[odi14023-bib-0033] World Health Organization (WHO) . (2020). Life expectancy at birth. Retrieved August 30, 2020, from https://www.who.int/data/gho/data/indicators/indicator‐details/GHO/life‐expectancy‐at‐birth‐(years

