# Supplementary figures and images for: Relation between number of teeth, malnutrition, and 3‐year mortality in elderly individuals ≥85 years
Source: Oral Dis. 2021 Sep 27;29(2):827–35. doi: 10.1111/odi.14023 (PMC10078753; doi:10.1111/odi.14023)

Figure S1

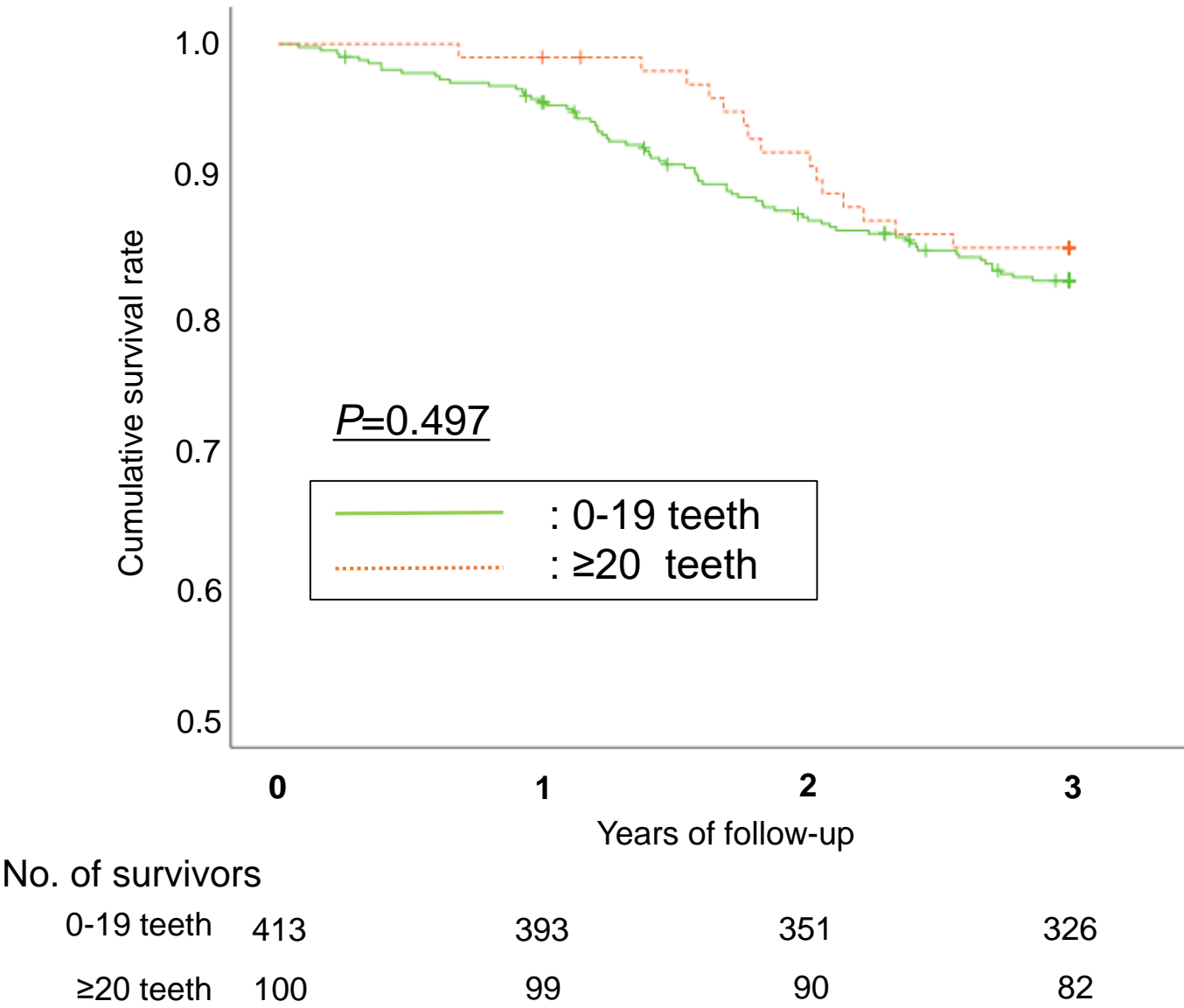

Supplement: Supplementary file 1 — Fig S1 [file ODI-29-827-s001.pdf]
