# Supplementary material for: Relation between number of teeth, malnutrition, and 3‐year mortality in elderly individuals ≥85 years
Source: Oral Dis. 2021 Sep 27;29(2):827–35. doi: 10.1111/odi.14023 (PMC10078753; doi:10.1111/odi.14023)
Supplement: Supplementary file 2 — Table S1‐3 [file ODI-29-827-s002.docx]

**Appendix : Table S1** Assessment criteria of GLIM

| **Phenotypic** | |  |  |  |  |  |  |  |  |  |
| --- | --- | --- | --- | --- | --- | --- | --- | --- | --- | --- |
| 1: Non-volitional weight loss | | |  |  |  |  |  |  |  |  |
| Weight loss ≥ 3 kg in a year | | |  |  |  |  |  |  |  |  |
| 2: Low body mass index | | |  |  |  |  |  |  |  |  |
| Body mass index < 20 (for Asian, over 70s) | | | |  |  |  |  |  |  |  |
| 3: Reduced muscle mass | | |  |  |  |  |  |  |  |  |
| Calf Circumference, male: < 34 cm, female: < 33 cm | | | | | | |  |  |  |  |
|  |  |  |  |  |  |  |  |  |  |  |
| **Etiologic** |  |  |  |  |  |  |  |  |  |  |
| 4: Reduced food intake or assimilation | | | |  |  |  |  |  |  |  |
| GOHAI question 1 (How often do you limit the kinds or amounts of food you eat because of problems with your oral condition?) | | | | | | | | | | |
| The answer of “always” and “often” demonstrated reduction in food intake. | | | | | | | |  |  |  |
| 5: Disease burden/inflammation | | | |  |  |  |  |  |  |  |
| C-reactive protein (>0.3 mg/dl) | | | | |  |  |  |  |  |  |
|  |  |  |  |  |  |  |  |  |  |  |
| Diagnosis of malnutrition requires at least 1 Phenotypic criterion and 1 Etiologic criterion | | | | | | | | |  |  |

**Abbreviations:** GOHAI, Geriatric Oral Health Assessment Index; GLIM, Global Leadership Initiative on Malnutrition

**Appendix : Table S2** Hazard ratios for the presence or absence of malnutrition

|  | Model 4 (n = 458) | | |  | Model 5 (n = 489) | | |
| --- | --- | --- | --- | --- | --- | --- | --- |
|  | HR | 95 ％ Cl | *p*-value |  | HR | 95 ％ Cl | *p*-value |
| malnutrition | 2.166 | [1.267–3.702] | 0.005 |  | 2.289 | [1.400–3.743] | 0.001 |

**Abbreviations:** HR, hazard ratio; Cl, confidence interval; BMI, body mass index; MMSE, Mini-Mental State Examination; MOF, Maximam Occulusal Force; GOHAI, Geriatric Oral Health Assessment Index

**Notes:** Model 2: Adjusted for age, gender, living alone, MMSE, handgrip strength, and medical history (cancer and diabetes).

Model 4: Model 3: Adjusted for Model 2 and MOF, Model 5: Adjusted for Model 2 and GOHAI

**Appendix : Table S3 Relation between Eichner Index and malnutrition**

|  | Eichner Index | | | |
| --- | --- | --- | --- | --- |
|  | A | B | C | *P** |
| Malunutrition (％) | 18 | 14 | 19 | 0.569 |

**Notes:** A, Occlusal contacts are present in all four posterior support area. B, occlusal contacts are present in 1–3 area of contacts or in the anterior region only. C, 0 occlusal contacts. The number of participant are as below; A 49, B 112, C 343.

*P*-value was calculated using the chi-square test.

**Appendix : FigureS1** Number of teeth and 3-year all-cause mortality. Kaplan–Meier survival curves for the two categories (0–19, ≥20) of number of teeth (log-rank *P* = 0.497). Cumulative survival rates did not differ due to differences in the number of teeth.
