# Supplementary material for: Relation between number of teeth, malnutrition, and 3‐year mortality in elderly individuals ≥85 years
Source: Oral Dis. 2021 Sep 27;29(2):827–35. doi: 10.1111/odi.14023 (PMC10078753; doi:10.1111/odi.14023)
Supplement: Supplementary file 3 — Supplementary Material [file ODI-29-827-s003.doc]

Appendix : STROBE Statement—checklist of items that should be included in reports of observational studies

|  | Item No | Recommendation |
| --- | --- | --- |
| **Title and abstract** | 1 | (*a*) Indicate the study’s design with a commonly used term in the title or the abstract  (lines 39–47) |
|  |  | (*b*) Provide in the abstract an informative and balanced summary of what was done and what was found (lines39–54) |
| Introduction | | |
| Background/rationale | 2 | Explain the scientific background and rationale for the investigation being reported  (lines58–81) |
| Objectives | 3 | State specific objectives, including any prespecified hypotheses (lines81–85) |
| Methods | | |
| Study design | 4 | Present key elements of study design early in the paper (lines 88–91) |
| Setting | 5 | Describe the setting, locations, and relevant dates, including periods of recruitment, exposure, follow-up, and data collection (lines 88–93 and 165–168) |
| Participants | 6 | (*a*) *Cohort study*—Give the eligibility criteria, and the sources and methods of selection of participants. Describe methods of follow-up (lines 91–95 and 165–168.  The summary of TOOTH study was reported by ref (Arai et al., 2010)). |
|  |  | (*b*) *Cohort study*—For matched studies, give matching criteria and number of exposed and unexposed (lines 91–95 and Table 1) |
| Variables | 7 | Clearly define all outcomes, exposures, predictors, potential confounders, and effect modifiers. Give diagnostic criteria, if applicable (lines 165–167 and 170–189,) |
| Data sources/ measurement | 8* | For each variable of interest, give sources of data and details of methods of assessment (measurement). Describe comparability of assessment methods if there is more than one group (lines 105–162) |
| Bias | 9 | Describe any efforts to address potential sources of bias (lines 199–201 in “RESULTS” section and lines 286–289 in the study limitation section) |
| Study size | 10 | Explain how the study size was arrived at (According to a previous publication (Arai et al, 2010)) |
| Quantitative variables | 11 | Explain how quantitative variables were handled in the analyses. If applicable, describe which groupings were chosen and why (lines 170–174) |
| Statistical methods | 12 | (*a*) Describe all statistical methods, including those used to control for confounding  (lines 170–189) |
|  |  | (*b*) Describe any methods used to examine subgroups and interactions (lines 211–214 in “RESULTS” section) |
|  |  | (*c*) Explain how missing data were addressed (lines 187 and 188 ) |
|  |  | (*d*) *Cohort study*—If applicable, explain how loss to follow-up was addressed  (lines 199–201 in “RESULTS” section)  *Case-control study*—If applicable, explain how matching of cases and controls was addressed  *Cross-sectional study*—If applicable, describe analytical methods taking account of sampling strategy |
|  |  | (*e*) Describe any sensitivity analyses NA |

Continued on next page

| Results | | |
| --- | --- | --- |
| Participants | 13* | (a) Report numbers of individuals at each stage of study—eg numbers potentially eligible, examined for eligibility, confirmed eligible, included in the study, completing follow-up, and analysed (Precisely described in the Participants section.) |
|  |  | (b) Give reasons for non-participation at each stage(According to a previous publication (Arai et al, 2010)) |
|  |  | (c) Consider use of a flow diagram (According to a previous publication (Arai et al, 2010)) |
| Descriptive data | 14* | (a) Give characteristics of study participants (eg demographic, clinical, social) and information on exposures and potential confounders (lines 191–197) |
|  |  | (b) Indicate number of participants with missing data for each variable of interest (Table 1) |
|  |  | (c) *Cohort study*—Summarise follow-up time (eg, average and total amount) (line 199) |
| Outcome data | 15* | *Cohort study*—Report numbers of outcome events or summary measures over time  (Figure1-3) |
|  |  | *Case-control study—*Report numbers in each exposure category, or summary measures of exposure |
|  |  | *Cross-sectional study—*Report numbers of outcome events or summary measures |
| Main results | 16 | (*a*) Give unadjusted estimates and, if applicable, confounder-adjusted estimates and their precision (eg, 95% confidence interval). Make clear which confounders were adjusted for and why they were included (lines 206–210) |
|  |  | (*b*) Report category boundaries when continuous variables were categorized (lines 191–192) |
|  |  | (*c*) If relevant, consider translating estimates of relative risk into absolute risk for a meaningful time period |
| Other analyses | 17 | Report other analyses done—eg analyses of subgroups and interactions, and sensitivity analyses (Appendix : Table S2,3 and Figure S1) |
| Discussion | | |
| Key results | 18 | Summarise key results with reference to study objectives (lines 216–220) |
| Limitations | 19 | Discuss limitations of the study, taking into account sources of potential bias or imprecision. Discuss both direction and magnitude of any potential bias (lines 283–307) |
| Interpretation | 20 | Give a cautious overall interpretation of results considering objectives, limitations, multiplicity of analyses, results from similar studies, and other relevant evidence (lines 307–317) |
| Generalisability | 21 | Discuss the generalisability (external validity) of the study results(lines 291–294) |
| Other information | | |
| Funding | 22 | Give the source of funding and the role of the funders for the present study and, if applicable, for the original study on which the present article is based (lines 31–36) |

*Give information separately for cases and controls in case-control studies and, if applicable, for exposed and unexposed groups in cohort and cross-sectional studies.

**Note:** An Explanation and Elaboration article discusses each checklist item and gives methodological background and published examples of transparent reporting. The STROBE checklist is best used in conjunction with this article (freely available on the Web sites of PLoS Medicine at http://www.plosmedicine.org/, Annals of Internal Medicine at http://www.annals.org/, and Epidemiology at http://www.epidem.com/). Information on the STROBE Initiative is available at www.strobe-statement.org.
